# A validated protocol for eDNA-based monitoring of within-species genetic diversity in a pond-breeding amphibian

**DOI:** 10.1038/s41598-023-31410-4

**Published:** 2023-03-16

**Authors:** Lucia Zanovello, Matteo Girardi, Alexis Marchesini, Giulio Galla, Stefano Casari, Diego Micheletti, Sonia Endrizzi, Chiara Fedrigotti, Paolo Pedrini, Giorgio Bertorelle, Heidi Christine Hauffe

**Affiliations:** 1 Conservation Genomics Research Unit, Research and Innovation Centre, Fondazione Edmund Mach, San Michele all’Adige, TN Italy; 2 Conservation Biology Unit, MUSE - Science Museum Trento, Trento, Italy; 3 Department of Life Sciences and Biotechnology, University of Ferrara, Ferrara, Italy; 4 Institute for Sustainable Plant Protection (IPSP), The National Research Council of Italy (CNR), Sesto Fiorentino, Florence, Italy; 5 Computational Biology Research Unit, Research and Innovation Centre, Fondazione Edmund Mach, San Michele all’Adige, TN Italy; 6 National Biodiversity Future Center, S.c.a.r.l., Palermo, Italy

**Keywords:** Biological techniques, Genetics, Molecular biology, Zoology

## Abstract

In light of the dramatic decline in amphibian biodiversity, new cost-efficient tools to rapidly monitor species abundance and population genetic diversity in space and time are urgently needed. It has been amply demonstrated that the use of environmental DNA (eDNA) for single-species detection and characterization of community composition can increase the precision of amphibian monitoring compared to traditional (observational) approaches. However, it has been suggested that the efficiency and accuracy of the eDNA approach could be further improved by more timely sampling; in addition, the quality of genetic diversity data derived from the same DNA has been confirmed in other vertebrate taxa, but not amphibians. Given the availability of previous tissue-based genetic data, here we use the common frog *Rana temporaria* Linnaeus, 1758 as our target species and an improved eDNA protocol to: (i) investigate differences in species detection between three developmental stages in various freshwater environments; and (ii) study the diversity of mitochondrial DNA (mtDNA) haplotypes detected in eDNA (water) samples, by amplifying a specific fragment of the COI gene (331 base pairs, bp) commonly used as a barcode. Our protocol proved to be a reliable tool for monitoring population genetic diversity of this species, and could be a valuable addition to amphibian conservation and wetland management.

## Introduction

Of all taxa affected by the current biodiversity crisis, amphibians are the most endangered group of vertebrates^1^, with 41% of globally-evaluated IUCN species included in ‘threatened’ categories (IUCN, 2021). Due to the general elusiveness of amphibian adults and limited detectability of early life stages, which often live in protected, difficult-to-access and/or season-dependent aquatic environments, accurate estimates of the presence and distribution of amphibian species are logistically difficult, costly and time-consuming to obtain^2,3^.

In addition to species richness, within-species genetic diversity is of crucial importance for the persistence and evolution of natural populations^4,5^, enabling them to adapt to environmental changes, and to the spread of new pathogens^6,7^. Moreover, the loss of genetic variability may have detrimental effects on individual health due to inbreeding depression, reducing fitness and ultimately increasing the risk of population and species extinction^8^. Due to their particular breeding strategy (often r-strategists with small effective population sizes and high clutch mortality), low dispersal rates and high philopatry, all of which limit population connectivity, amphibians seem to be especially prone to genetic erosion^9^. Therefore, regular, cost-effective genetic monitoring should be considered a fundamental aspect of amphibian conservation strategies. Although the importance of protecting genetic diversity is widely recognized and has been addressed under the Aichi Biodiversity Targets^10^, the development of standardized monitoring frameworks for an accurate surveillance of genetic diversity trends in natural populations is still limited to few charismatic or economically relevant species^5,11^.

For the last decade, environmental DNA (i.e. DNA that can be extracted from noninvasive samples such as soil, water, fecal pellets, hair or feathers; eDNA) has been used for accurate, cost-efficient species detection in aquatic environments^12,13^, including for amphibians (for a recent review, see^3^). Specifically, single species eDNA surveys (mainly using quantitative PCR; qPCR) have increased the speed and efficiency of amphibian detection, compared to traditional observational monitoring^14,15^, and have proven to be particularly useful for detecting rare or elusive amphibian species (e.g.,^16–18^), improving the knowledge of habitat requirements and species distributions (e.g.^19–21^), and tracking invasive alien amphibians (e.g.^22–24^.). In addition, the eDNA metabarcoding approach is increasingly used as a cost-effective method for the simultaneous assessment of species composition in amphibian communities^3,25^. However, to our knowledge, only one study aiming at the development of a long-term eDNA-based monitoring program for an amphibian species has been published thus far^26^.

Very recently, the possibility of inferring intraspecific genetic diversity from eDNA has also been explored^3,27–29^; however, only a few pilot studies on amphibians have been published. For example, Gorički et al.^30^ used two short CytB fragments (about 100–150 bp) for the discrimination of two putative olm (*Proteus anguinus)* subspecies, but the protocol was not designed to estimate population-level genetic diversity. Wang et al.^31^ developed an eDNA metabarcoding protocol for the Chinese giant salamander (*Andrias davidianus*) to allow the detection of seven haplotypes corresponding to distinct evolutionary lineages (reporting the results of laboratory amplicon mixture), but again the study was not aimed at the assessment of within-population genetic variability.

Despite their great potential, eDNA methods are extremely sensitive to sampling design and field and laboratory protocols, as eDNA is not abundant and persists in aquatic environments for a limited time, from a few days to several weeks^32–34^. Therefore, developing and validating effective field sampling methods is essential to the application of eDNA-based survey methods^35–37^.

In this study, we chose the common frog (*Rana temporaria* Linnaeus, 1758), a widespread European pond-breeding amphibian^38^, showing high genetic diversity^39,40^, to develop and validate an eDNA metabarcoding protocol allowing rapid and standardized assessments of within-population genetic variability from water samples. Specifically, by selecting 10 wetland sites, for which we had previous information on *R. temporaria* haplotypes identified using traditional tissue sampling^41^, we: (a) developed a metabarcoding protocol targeting a 331 bp long fragment of the COI region, which allowed discrimination of previously identified haplotypes; (b) optimized the sampling design, in terms of temporal and spatial replicates, for the collection of water samples in a variety of wetland habitats; (c) computed standard genetic diversity estimates for *R. temporaria* populations from eDNA metabarcoding, to compare eDNA-based results with previously available genetic data.

## Materials and methods

### Study species and study area

The common frog has been a model species for previous genetic diversity studies (e.g.,^39,40^); however, despite being the most widespread amphibian species in Europe^42^, local population declines are frequent due to climate change and habitat degradation^43,44^. Our study area covers the Autonomous Province of Trento (Italy), a mountainous region located in the eastern Alps; mtDNA haplotypes data are available for *R. temporaria* across the study area from a recent genetic survey^41^.

### Sample collection

**Figure 1:**
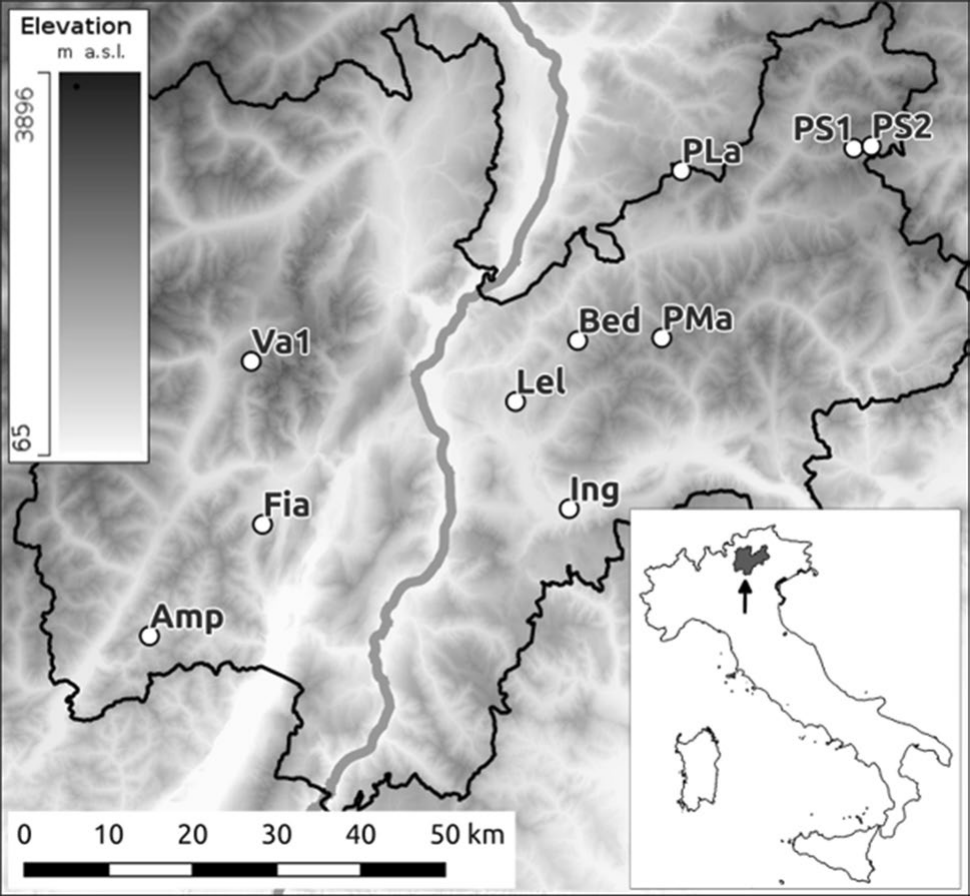
Map of the study region (Province of Trento, Italy) showing the 10 selected wetland sample sites, labeled according to Table 1. Site locations, abbreviations, coordinates, elevation and description are listed in Supplementary Information, Table S1. The map was generated using QGIS version 3.20 (http://www.qgis.org); Digital Terrain Model (DTM) for the study area was extracted from the 20 m-resolution DTM of Italy, publicly available at the National Geoportal of Italy (http://www.pcn.minambiente.it/mattm/servizio-di-scaricamento-wcs/).

For the present study, 10 sites were selected from Marchesini et al.^41^ (Fig. 1 and Supplementary Information, Table S1) that encompassed all three mitochondrial lineages of *R. temporaria* known to be present in the study area, as well as the majority of the haplotypes (8/12) noted in^41^.

Water samples were collected at each of the 10 sites during the reproductive season (March-September depending on altitude) from 2019 to 2021. In order to define a standard protocol, three temporal (T1-T3) and three spatial (S1-S3) replicates were collected, where T1 corresponds to a replicate collected when eggs or early-stage larvae were present at the site (early spring), T2, when late-stage larvae were present (mid-summer), and T3, when both adults and juveniles had abandoned the sites following reproduction (end of summer/ early autumn). Some of the wetland sites became partially desiccated during the study, thus collecting the T3 replicates was not always possible. When possible (i.e. whenever spatially separated, accessible water microhabitats were available at the site), for each temporal replicate, three spatial replicates were sampled (5/10 sites) close to observed life stages. For three of these sites, for one of the three spatial replicates (Amp-T1-S2; Ing-T1-S3; PMa-T1-S1, Table 1 and in Supplementary Information, Table S2), no egg clutches or tadpoles were detected at T1; nonetheless, sampling was performed in any case, and all sampled microhabitats were included in the experimental design. In the remaining five sites, only two spatial replicates were possible. In order to capture as many haplotypes as possible from the same sampling site, the spatial replicates were collected at least 20 m apart (depending on the area of wetland on the sampling site).

**Table 1:** Spatial and temporal replicates included in the eDNA metabarcoding study of *Rana temporaria* in the Province of Trento, Italy.

Site	T1				T2				T3				
	S1	S2	S3	Repl. T1	S1	S2	s3	Repl. T2	S1	S2	S3	Repl. T3	Tot Repl
Amp	x	nd	x	2/3	nd	x	x	2/3	nd	nd	nd	0/3	4/9
Bed	x	x	/	2/2	x	x	/	2/2	nd	nd	/	0/2	4/6
Fia	x	x	x	3/3	x	x	nd	2/3	nd	nd	nd	0/3	5/9
Ing	x	x	nd	2/3	x	nd	nd	1/3	nd	nd	nd	0/3	3/9
Lel	x	nd	/	1/2	nd	/	/	0/1	/	/	/	0/0	1/3
Pla	x	x	x	3/3	nd	nd	nd	0/3	nd	nd	nd	0/3	3/9
PMa	nd	x	x	2/3	nd	nd	nd	0/3	nd	nd	nd	0/3	2/9
PS1	x	x	/	2/2	x	nd	/	1/2	nd	nd	/	0/2	3/6
PS2	x	x	/	2/2	nd	nd	/	0/2	nd	nd	/	0/2	2/6
Va1	x	x	/	2/2	x	x	/	2/2	x	nd	/	1/2	5/6
												TOT	32/72

T1, T2, T3 = temporal replicates 1, 2, and 3; Repl. T1, Repl. T2, Repl. T3: for every temporal point, the sum of spatial replicates per site is reported (positive/total); S1, S2, S3 = spatial replicates 1, 2, and 3; x = sampling was performed and positive results were obtained after sequencing; nd = sampling was performed but amplification was not successful (no DNA bands visible on the Qiaxcel Advanced System (Qiagen) and/or several samples were sequenced for confirmation and no *R. temporaria* DNA was detected); / = sampling was not performed because the structure of the wetland site was not compatible with three spatial replicates (see text for details) or, (for T3) one of the previously selected water microhabitats was no longer physically accessible or non-existent (due to seasonal changes in wetland extent and hydrology).

Each replicate sample was collected manually from just under the surface of the water body using a sterile plastic canister. Stirring up sediment was carefully avoided. To minimize sample contamination, field workers wore laboratory gloves (changed between each replicate), collected samples from the shore without disturbing the water body, and wiped all equipment with bleach and alcohol between each site. The water collected in the canister was stirred before being drawn up with a 100 ml syringe and filtered through a Sterivex-GP Filter unit (pore size 0.22 µm, Millipore cat. no. SVGPL10RC); this step was repeated until the filter clogged. A second filter was also used for the same spatial replicate. The quantity of water that could be filtered through two Sterivex-GP Filter units varied widely among sites, ranging from about 100 ml to more than one liter, depending on the suspended organic matter. Each filter was drained and capped at both ends with the inlet and outlet caps. Following the manufacturer’s instructions, all filters were kept at ambient temperature out of direct sunlight until arrival in the laboratory later the same day, and archived at − 20 °C until DNA extraction.

### Sample processing, including primer validation

All laboratory procedures were carried out at the Platform of Animal, Environmental and Antique DNA of the Conservation Genomics Research Unit, Fondazione Edmund Mach, following recommended guidelines for eDNA analyses, including separate pre- and post-PCR laboratories^45,46^. All procedures were performed under BSL2 biological hoods. DNA extraction from filters was carried out using the DNeasy PowerWater Sterivex Kit (Qiagen), following the manufacturer’s instructions with these modifications: the heating step and bead beating tubes (PowerBead Tubes) were eliminated to reduce extraction of non-target genetic material from microorganisms. The two filters corresponding to the same spatial replicate S1, S2, or S3 were processed simultaneously and extracts were merged into a single tube at the 14th step of the protocol. DNA extraction was performed in batches of a maximum of 10 water replicate samples, including one negative control (extraction blank) for each extraction batch.

To design a primer pair for amplifying the diagnostic mtDNA region for *R. temporaria,* Primer3Plus^47^ was used with the haplotype sequences available from Marchesini et al.^41^. The resulting primer pair, named Rt-aplo_COI F (GTAATAATTTTCTTTATGGT) and Rt-aplo_COI R (TCAAACAAAGAGGGGTGT), amplifies a fragment 331 bp long distinguishing 11 of the 12 mtDNA haplotypes listed in Marchesini et al.^41^ for the Province of Trento. Specifically, haplotype pairs CA2 and TN4, and SA1 and PR7 (the latter not being previously found in the study area) were not distinguishable, and therefore, were named CA2_TN4 and SA1_PR7, respectively, in the Results section. To distinguish all known haplotypes for the study area, a 569 bp sequence plus adapters (thus about 690 bp in total) would have been required, but such a long fragment cannot be processed using the Illumina technology commonly adopted for metabarcoding (maximum length 600 bp). The primer pair was tested in vitro on DNA extracted from *R. temporaria* tissue samples available from previous studies, and amplification success of all samples was confirmed via screening on a Qiaxcel Advanced System (Qiagen). The amplification reaction took place in a final volume of 50µL, containing H_2_O (22.25µL), Promega Flexi Buffer 5X (10µL), MgCl_2_ 25 mM (4µL), BSA 10 mg/mL (0.5µL), Rt-aplo_COI-F 10 ρmol/μL (1µL) and Rt-aplo_COI-R 10 ρmol/μL (1µL), dNTP’s 10 mM each (1µL total), Promega GoTaq G2 5U/µL (0.25µL) and template DNA (10µL). The PCR mixture was denatured at 95 °C for 2 min, followed by 40 cycles of 30 s at 95 °C, 15 s at 48 °C and 40 s at 72 °C, and a final elongation step at 72 °C for 5 min. Only one PCR was run per replicate, and a second PCR was run only if the first one failed to produce results. One negative control (PCR blank) was included for each PCR reaction, along with all extraction negative controls.

Each amplification product was then purified with the MinElute PCR Purification Kit (Qiagen) following manufacturer’s instructions. 20 µl of each purified product were loaded into a single 96-well plate and sequenced at the FEM Sequencing and Genotyping Platform using paired-end sequencing (2 × 300 bp) on an Illumina Miseq (Illumina, San Diego, CA) with a 30 000 bp coverage.

### Bioinformatics and statistical analysis

All bioinformatic analyses were performed with the software MICCA^48^. Overlapping paired-end sequences were merged to obtain consensus sequences using the command ‘mergepairs’ with a minimum overlap length of 100 bp and maximum of one mismatch in the overlap region. Reads that did not contain the forward or reverse primers were discarded, and primers were trimmed from the remaining fragments with the command ‘trim’. Sequences were quality filtered using the command ‘filter’, assuming a maximum allowed expected error rate of 0.1% and a minimum length of 331 bp (exact length of the target sequence without primers, as is becoming common practice for genetic diversity studies based on eDNA metabarcoding, e.g.,^27,49^). The reference database for haplotype classification was assembled using published *R. temporaria* haplotype sequences found in Italy. The method ‘otu open_ref’ was used to cluster our sequences against this database with an identity threshold of 0.99, rejecting a sequence if the fraction of alignment to the reference sequence was lower than one, and discarding sequences with a read abundance value lower than 100 after dereplication. To avoid false positives, either from PCR or sequencing errors, or minor cross-contaminations between the replicate samples, OTUs represented by fewer reads than 5% of the total reads for a specific replicate sample were discarded^27^.

To calculate the diversity indices, haplotype relative frequencies were obtained across spatial and temporal replicates through simulated datasets created with a Linux shell script. The datasets were built such as that, for each site and each recorded haplotype, the haplotypes frequencies were equal to the haplotype total number of reads (sequence counts) divided by 100. The simulated datasets thus represent hypothetical populations in which absolute abundance (in terms of sequence counts) of each haplotype ideally correspond to the number of individuals carrying the haplotype. As the abundances calculated from eDNA data could be influenced by several limitations (i.e. non-exhaustive sampling in terms of spatial coverage of the site, potential preferential amplification between different haplotypes, potential differences in DNA particles release from different coexisting life stages of the species), these estimates may not be as accurate as more invasive methods. For a more precise estimate of the population genetic diversity, a traditional sampling is still desirable. However, the reproductive behavior of *R. temporaria*, similar to other pond-breeding amphibians, is characterized by an ‘explosive’ reproduction, producing large numbers of egg masses sometimes crowded together. Furthermore, in our target species, generally one female lies one egg clutch, therefore the number of egg clutches roughly corresponds to the number of females in the population. Therefore, as eDNA samples were collected when larvae/eggs presence was visually confirmed, and by sampling multiple spatial replicates, the chances of obtaining a sufficiently representative sampling of the population’s gene pool were increased.

Standard diversity indices were calculated for each site (number of haplotypes, n; haplotype diversity, h; nucleotide diversity, π) using the software DnaSP v6^50^, and Spearman rank correlations tests were performed using RStudio to compare these estimates with those reported in Marchesini et al.^41^. Since the estimates of haplotype frequencies and diversity indices from the two methods are not exactly the same (i.e. the tissue based measures were based on the number of individuals in which a haplotype was found, whereas eDNA haplotype frequencies and diversity were based on the number of samples through space in which each haplotype was detected), non-metric multidimensional scaling (NMDS), based on Bray–Curtis dissimilarity matrices and computed using the R package ‘vegan’ 2.6–4, was used to graphically represent differences between the eDNA metabarcoding and the reference datasets; these two datasets were then statistically compared with a Mantel test with 10000 permutations in RStudio. A correlation between the two measures was expected if eDNA was capable of detecting nearly all haplotypes (since common haplotypes would also be more widespread).

## Results

A total of 72 water samples were collected and filtered at the 10 sampling sites. *Rana temporaria* DNA was successfully amplified from 32 of these. T1 replicates were the most successful, with 21/25 eDNA-positive replicate samples, while T2 replicates yielded results in 10/24 replicates (Table 1). T3 replicates did not produce positive results for any sites except Va1 (data not shown). All negative controls from both the extraction and PCR steps were considered not contaminated, as none had more than 10 reads; therefore, these were removed during the OTU clustering step. The spatial replicates showed high variability in terms of amplification success, identified haplotypes and their relative frequencies for each sampling site (Tables 1 and 2).

**Table 2:** COI haplotype frequencies for *Rana temporaria* populations in the Province of Trento detected by eDNA metabarcoding across temporal replicates. For each wetland site, haplotype frequencies (mean of spatial replicates S1-3) detected by eDNA metabarcoding at the different temporal points (T1, T2 and mean T1 + T2) are reported, together with available frequencies derived from traditional tissue-based genetic sampling (reference dataset: ^41^). nd = no data.

Site	Dataset	COI haplotypes (frequencies)							
		CA2_TN4	VC6	PR4	SA1_PR7	TN2	TN3	TN5	MT5
Amp	eDNA (T1)	0.644	0	0	0	0.74	0	0	0
	eDNA (T2)	0.317	0	0	0	0.68	0	0	0
	eDNA (T1 + T2)	0.2907	0	0	0	0.71	0	0	0
	reference dataset	0.4	0	0	0	0.6	0	0	0
Bed	eDNA (T1)	0.2637	0.61	0.122	0	0	0	0	0
	eDNA (T2)	0.2006	0.59	0.209	0	0	0	0	0
	eDNA (T1 + T2)	0.2321	0.6	0.166	0	0	0	0	0
	reference dataset	0.1	0.8	0.1	0	0	0	0	0
Fia	eDNA (T1)	0.3871	0	0	0	0.16	0.43	0.0196	0
	eDNA (T2)	0.4415	0	0	0	0.06	0.49	0	0
	eDNA (T1 + T2)	0.4089	0	0	0	0.12	0.46	0.0118	0
	reference dataset	0.3	0	0	0	0.1	0.3	0.3	0
Ing	eDNA (T1)	0.9528	0.05	0	0	0	0	0	0
	eDNA (T2)	0.2345	0.77	0	0	0	0	0	0
	eDNA (T1 + T2)	0.7133	0.29	0	0	0	0	0	0
	reference dataset	0.8	0.2	0	0	0	0	0	0
Lel	eDNA (T1)	0.2203	0.78	0	0	0	0	0	0
	eDNA (T2)	nd	nd	nd	nd	nd	nd	nd	nd
	eDNA (T1 + T2)	0.2203	0.78	0	0	0	0	0	0
	reference dataset	0.5	0.4	0	0.1^a^	0	0	0	0
PLa	eDNA (T1)	0.8031	0	0.134^b^	0.0628	0	0	0	0
	eDNA (T2)	nd	nd	nd	nd	nd	nd	nd	nd
	eDNA (T1 + T2)	0.8031	0	0.134^b^	0.0628	0	0	0	0
	reference dataset	0.8	0	0	0.2	0	0	0	0
PMa	eDNA (T1)	0.1754	0.82	0	0	0	0	0	0
	eDNA (T2)	nd	nd	nd	nd	nd	nd	nd	nd
	eDNA (T1 + T2)	0.1754	0.82	0	0	0	0	0	0
	reference dataset	0.7	0.3	0	0	0	0	0	0
PS1	eDNA (T1)	0.3936	0.35	0.26	0	0	0	0	0
	eDNA (T2)	0.7635	0	0.237	0	0	0	0	0
	eDNA (T1 + T2)	0.5169	0.23	0.252	0	0	0	0	0
	reference dataset	0.3	0.2	0.5	0	0	0	0	0
PS2	eDNA (T1)	0.6715	0.09	0.238	0	0	0	0	0
	eDNA (T2)	nd	nd	nd	nd	nd	nd	nd	nd
	eDNA (T1 + T2)	0.6715	0.09	0.238	0	0	0	0	0
	reference dataset	0.5	0.1	0.4	0	0	0	0	0
Va1	eDNA (T1)	0.5235	0	0	0	0.24	0.19	0.0373^b^	0
	eDNA (T2)	0.4887	0	0	0	0.1	0.25	0.1544^b^	0
	eDNA (T1 + T2)	0.5061	0	0	0	0.17	0.22	0.0959^b^	0
	reference dataset	0.4	0	0	0	0.1	0.2	0	0.3^a^

^a^undetected by eDNA metabarcoding, but present in the reference dataset^41^; ^b^Detected by eDNA metabarcoding but not in the reference dataset^41^.

**Figure 2:**
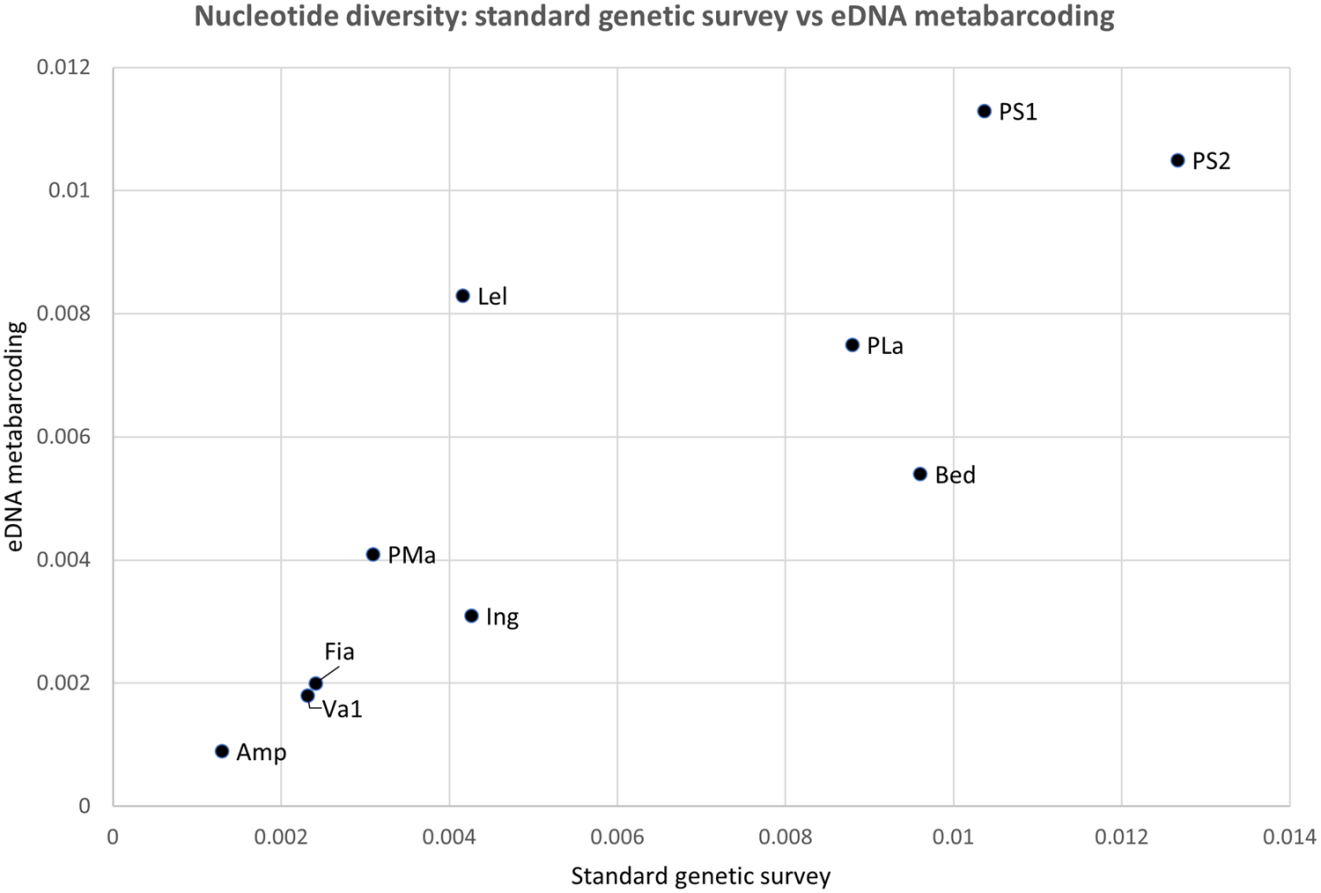
Scatterplot of the correlation between the nucleotide diversity index values for *Rana temporaria* in the Province of Trento Italy, for two datasets: x-axis: standard tissue-based genetic survey ^41^; y-axis: current study (eDNA metabarcoding).

**Figure 3:**
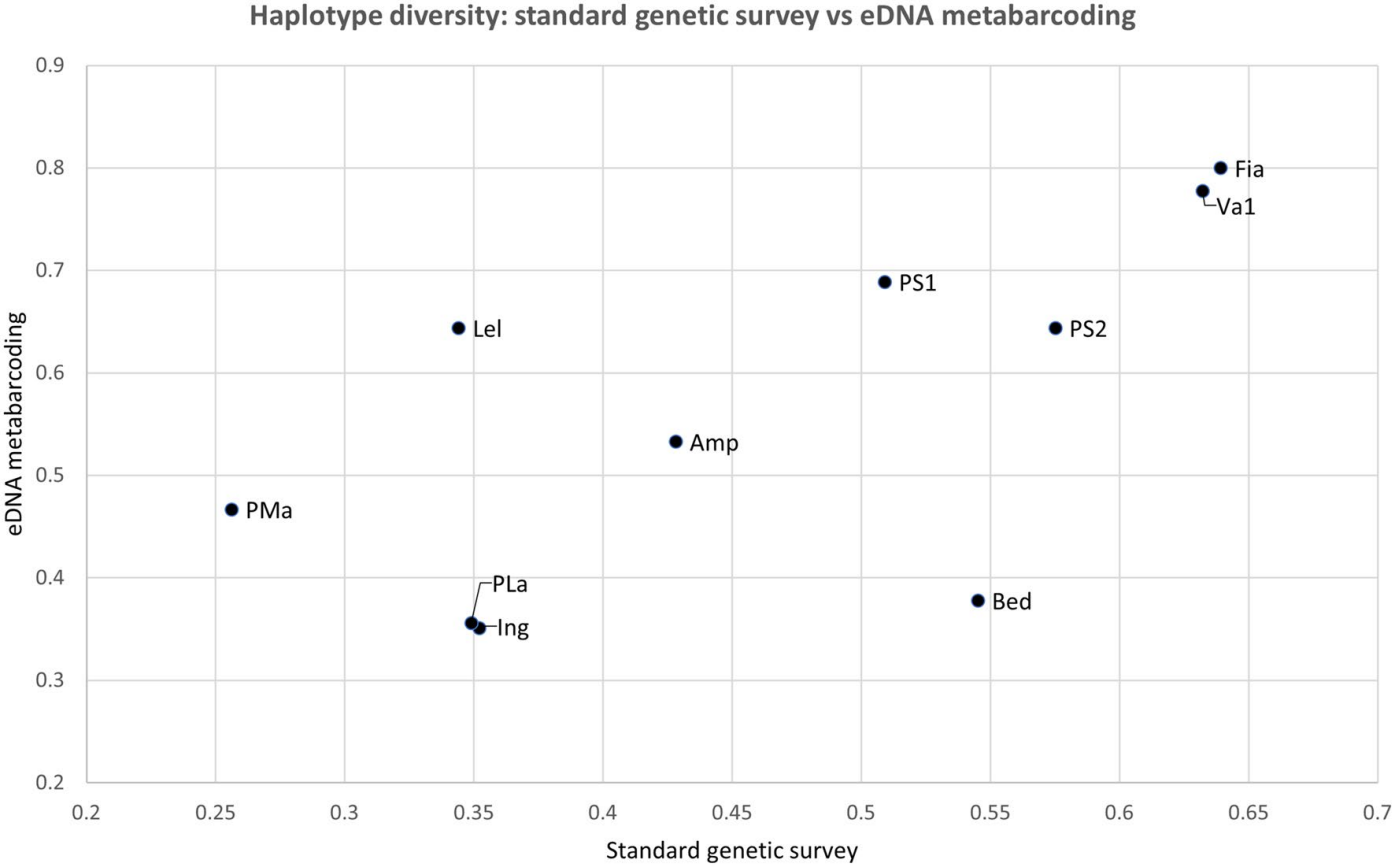
Scatterplot of the correlation between the haplotype diversity index values for *Rana temporaria* in the Province of Trento Italy, for two datasets: x-axis: standard tissue-based genetic survey ^41^; y-axis: current study (eDNA metabarcoding).

Ten COI haplotypes (including CA2_TN4 and SA1_Pr7) were detected, all belonging to the three Alpine lineages already known to be present in the province of Trento^41^. The genetic diversity estimates calculated from the eDNA dataset are reported in Table 3. Considering the haplotype detected in all replicates from each site, the number of haplotypes detected with our eDNA protocol showed a strong and statistically significant correlation with the number of haplotypes found in the previous study (R = 0.78, *p *= 0.008). For eight out of 10 sites, in fact, the number of haplotypes detected by the two approaches was the same. The remaining two sites only differed for one haplotype. Similarly, nucleotide diversity for the eDNA data was strongly correlated with π of the reference dataset (R = 0.88, *p *= 0.002), as shown in Fig. 2. Haplotype diversity (h) from the two datasets (Fig. 3) showed a more moderate but statistically significant correlation (R = 0.63, *p *= 0.05). If only T1 (the temporal replicate with the highest number of positive samples) was considered, the correlation between the number of haplotypes calculated with the two datasets remained unchanged (R = 0.78, *p *= 0.008), while both h and π estimated from eDNA data showed a slightly higher correlation with the same indices from the reference dataset (R = 0.74, *p *= 0.014 and R = 0.9, *p * = 0.001, respectively).

**Table 3:** Genetic diversity estimates for *Rana temporaria* populations in the Province of Trento: results of eDNA metabarcoding from the present study compared to the reference dataset^41^.

Site	No haplotypes (n)			Haplotype div. (h)			Nucleotide div. (pi)		
	eDNA (T1)	eDNA (T1 + T2)	tissue DNA	eDNA (T1)	eDNA (T1 + T2)	DNA	eDNA (T1)	eDNA (T1 + T2)	tissue DNA
Amp	2	2	2	0.368	0.428	0.533	0.001	0.001	9E-04
Bed	3	3	3	0.543	0.545	0.378	0.009	0.01	0.005
Fia	4	4	4	0.644	0.639	0.8	0.002	0.002	0.002
Ing	2	2	2	0.121	0.352	0.351	0.001	0.004	0.003
Lel	2	2	3	0.344	0.344	0.644	0.004	0.004	0.008
PLa	3	3	2	0.349	0.349	0.356	0.009	0.009	0.008
PMa	2	2	2	0.256	0.256	0.467	0.003	0.003	0.004
PS1	3	3	3	0.533	0.509	0.689	0.011	0.01	0.011
PS2	3	3	3	0.575	0.575	0.644	0.013	0.013	0.011
Va1	4	4	4	0.601	0.632	0.778	0.002	0.002	0.002

eDNA (T1): eDNA metabarcoding considering only T1 replicates; eDNA (T1 + T2): eDNA metabarcoding considering T1 + T2 replicates; tissue DNA: estimates from the reference dataset^41^.

**Figure 4:**
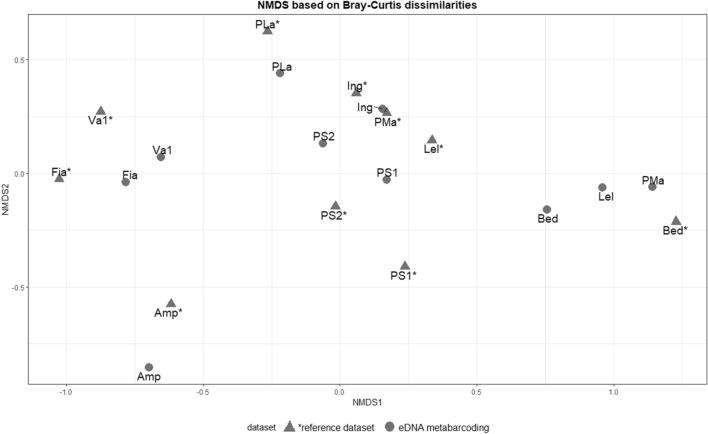
Non-metric multidimensional scaling (NMDS) plot, based on two Bray- Curtis dissimilarity matrices for *Rana temporaria* in the Province of Trento Italy, for two datasets: sites represented by a triangle and labeled with ‘*’ refer to the standard genetic survey dataset ^41^; sites represented with a circle and without ‘*’ refer to the eDNA metabarcoding dataset.

The NMDS plot (shown in Fig. 4) showed that, for each sampling site, the eDNA metabarcoding datapoints were closer to the reference datapoints from the same site, than to those of other sites, suggesting a reasonable match between eDNA metabarcoding results and the reference dataset, except for two sites (i.e.: Lel, PMa). Overall, the Mantel test consistently showed a statistically significant correlation between population genetic dissimilarities based on eDNA metabarcoding data and those based on the reference dataset (R = 0.44, *p *= 0.005). In addition, the NMDS plot showed that three sites (Amp, Fia and Va1) cluster closely together with respect to the remaining seven, confirming the previously known distribution of *R. temporaria* haplotypes; that is, two clusters, one in western and one in eastern Trentino^41^.

## Discussion

In this study, all statistical tests showed that our eDNA metabarcoding protocol targeting a 331 bp long fragment of the COI region could identify haplotypes of *R. temporaria* previously identified from tissue samples, most reliably in the spring season. In addition, our protocol allowed us, for the first time, to provide an estimation of standard genetic diversity indices for an amphibian species from eDNA.

Our experimental design allowed us to identify an optimal sampling regime, in terms of temporal and spatial replicates, for the collection of eDNA water samples in a variety of small wetland habitats. Our results are in agreement with the recommendation from previous studies^51,52^ of sampling an adequate number of spatial replicates per site (up to one per 10–20 m perimeter), as this distribution of replicates allowed us to capture the full set of expected haplotypes from each sampling site. Regarding the temporal replicates, T1 alone allowed us to identify almost all haplotypes present per site in a given monitoring year. In fact, T1 yielded the same results, in terms of number of haplotypes identified, as those attained from the union of temporal replicates 1 and 2 (while no T3 replicates yielded target species DNA). Thus, if a multi-year monitoring program is being considered, we suggest implementing a simplified version of our protocol, with T1 as the optimal time point, as a strategy to maximize the cost/benefit ratio. However, as the combination of T1 and T2 replicates provided the most accurate data on haplotypes frequencies, for a more complete view of *R. temporaria* genetic diversity we propose that the protocol should be applied at least every three to five years. Replicate T3 did not provide any information on species presence and genetic diversity, and should not be considered for future eDNA protocol applications for amphibians that have a reproductive cycle similar to that of *R. temporaria*.

All the standard genetic diversity estimates computed here (namely, the number of haplotypes, h and π) proved to be statistically correlated with the same indices computed in the reference dataset from Marchesini et al.^41^. In agreement with the NMDS plot (Fig. 4), the Mantel test shows a moderate, statistically significant, correlation between the dissimilarities computed based on eDNA metabarcoding data and those computed based on the reference dataset. Moreover, the NMDS plot based on eDNA data further confirms previous genetic distributions of *R. temporaria* haplotypes^41^, identifying the same two geographical clusters formed by the three sites Amp, Fia and Va1 (western Trentino) and the remaining seven (eastern Trentino).

Only three of the 10 sampling sites consistently showed different haplotype counts and frequencies between the two approaches, namely sites Lel, PLa and PMa (Laghestel, Passo Lavazé and Passo Manghen; Table 2 and Table S1 in Supplementary Information). Interestingly, in these sites, the target species is known to breed in small temporary ponds near a bigger pond (Lel), or in small alpine lakes (PLa, PMa)^53^. These habitats are especially prone to seasonal fluctuations in water availability; therefore, it is possible that the differences in the haplotypes detected with the two methods, as well as in their relative frequencies, might be due to the ephemeral nature and repeated recolonization of these three sampling areas. In fact, the influence of short-term climatic fluctuations on demographic and genetic characteristics of wild populations has been demonstrated recently^54^. Even so, the eDNA protocol was able to detect two haplotypes out of the three found at Lel in the reference dataset. In the case of PLa, the most abundant haplotype shows the same relative frequency according to both datasets, and our protocol was also able to detect the less common haplotype. In addition, our eDNA metabarcoding approach detected another haplotype, not previously reported for this site but known to be present in eastern Trentino (PR4, see Table 2). For PMa, the eDNA protocol identified the same haplotypes as the reference dataset, but frequencies were reversed (see Table 2). Finally, the differences between the two datasets could also be explained by fluctuations of allele frequencies over time (with samples collected in 2017 and 2021, respectively), and considering that the reference dataset did not necessarily represent the entire gene pool of the considered populations, being based on only 10 samples per site.

The protocol developed here, which is completely non-invasive and less time-consuming than traditional observational or tissue-based genetic surveys, could be implemented routinely in amphibian monitoring programs that integrate genetic diversity estimates with confirmation of the target species presence. We are currently improving and extending this holistic approach to all European amphibian species, but also to even rarer and more elusive invertebrates such as the European freshwater crayfish *Austropotamobius pallipes*. The same approach could also be used to determine the presence of invasive species, such as the American bullfrog *Lithobates catesbeianus*, which occurs in the regions surrounding our study site, but has not been reported thus far in the Province of Trento. Finally, we advocate the integration of nucleotide diversity estimates in eDNA-based approaches, included in very few studies thus far (e.g.,^28,29^, in order to obtain a detailed reconstruction of the potential adaptability of populations living in human- and/or climate change-impacted areas.

## Supplementary Information

Supplementary Information.

## Data Availability

Sequences available upon acceptance for publication of the manuscript or upon request. Correspondence and requests for materials should be addressed to H.C.H. (email: heidi.hauffe@fmach.it).
