# Supplementary material for: A validated protocol for eDNA-based monitoring of within-species genetic diversity in a pond-breeding amphibian
Source: Sci Rep. 2023 Mar 16;13:4346. doi: 10.1038/s41598-023-31410-4 (PMC10020426; doi:10.1038/s41598-023-31410-4)
Supplement: Supplementary file 1 — Supplementary Information. [file 41598_2023_31410_MOESM1_ESM.docx]

Table S1. Sampling sites with mean geographic coordinates (UTM 32N; units in m), mean elevation (m a.s.l.) and wetland type.

| **Sample Site** | **Abbrev** | **East/Long** | **West/Lat** | **Elevation (m a.s.l.)** | **Site description** |
| --- | --- | --- | --- | --- | --- |
| Lago d'Ampola | Amp | 628457 | 5081277 | 795 | small alpine lake with surrounding marshes |
| Bedollo | Bed | 679355 | 5116467 | 1183 | small alpine lake with surrounding marshes |
| Torbiera di Fiavé | Fia | 641894 | 5094567 | 665 | peatbog with small ponds and surrounding marshes |
| Inghiaie | Ing | 678339 | 5096436 | 444 | peatbog with small ponds |
| Laghestel | Lel | 671966 | 5109197 | 876 | peatbog with small ponds |
| Passo Lavazé | PLa | 691672 | 5136691 | 1802 | peatbog with small seasonal ponds |
| Passo Manghen | PMa | 689343 | 5116779 | 2083 | small alpine lake with surrounding peatbogs |
| Passo S. Pellegrino 1 | PS1 | 712169 | 5139382 | 1838 | peatbog with seasonal ponds |
| Passo S. Pellegrino 2 | PS2 | 714246 | 5139634 | 1940 | small alpine lake and surrounding peatbogs |
| Valagola | Va1 | 640550 | 5113989 | 1689 | peatbog with seasonal ponds |
| Lomasona | Lom | 644431 | 5093917 | 510 | peatbog with seasonal ponds |

Table S2. *Rana temporaria* life stages and additional observations during sampling. For each spatial (S repl.) and temporal (T repl.) replicate, the presence of *R. temporaria* was recorded for the following life stages: freshly laid egg clutches (E1); egg clutches, hatching (E2); larvae, initial stages (lL1); larvae, intermediate and final stages (L2); neometamorphs (N); adults (A). For each replicate, the corresponding outcome of eDNA metabarcoding is also reported (eDNA outcome; no = no *R. temporaria* DNA was amplified; /= the replicate was not collected). Additional observations on changes in site extent due to water loss are reported as footnotes.

|  |  |  |  | *R. temporaria* **stages** | | | | | |
| --- | --- | --- | --- | --- | --- | --- | --- | --- | --- |
| **Site** | **T repl.** | **S repl.** | **eDNA outcome** | **E1** | **E2** | **L1** | **L2** | **N** | **A** |
| Amp | T1 | S1 | yes |  |  | x |  |  |  |
| Amp | T1 | S2 | no |  |  |  |  |  |  |
| Amp | T1 | S3 | yes | x | x | x |  |  |  |
| Amp^a^ | T2 | S1 | no |  |  |  |  |  |  |
| Amp | T2 | S2 | yes |  |  |  | x |  |  |
| Amp | T2 | S3 | yes |  |  |  | x |  |  |
| Bed | T1 | S1 | yes | x |  |  |  |  |  |
| Bed | T1 | S2 | yes | x |  |  |  |  |  |
| Bed | T2 | S1 | yes |  |  |  | x | x |  |
| Bed | T2 | S2 | yes |  |  |  | x | x |  |
| Fia | T1 | S1 | yes | x |  |  |  |  |  |
| Fia | T1 | S2 | yes | x |  |  |  |  |  |
| Fia | T1 | S3 | yes | x |  |  |  |  |  |
| Fia | T2 | S1 | yes |  |  | x | x |  |  |
| Fia | T2 | S2 | yes |  |  | x | x |  |  |
| Fia | T2 | S3 | no |  |  |  | x |  |  |
| Ing | T1 | S1 | yes |  | x | x |  |  |  |
| Ing | T1 | S2 | yes |  | x | x |  |  |  |
| Ing | T1 | S3 | no |  |  |  |  |  |  |
| Ing | T2 | S1 | yes |  |  |  | x |  |  |
| Ing | T2 | S2 | no |  |  |  |  |  |  |
| Ing^a^ | T2 | S3 | no |  |  |  |  |  |  |
| Lel | T1 | S1 | yes | x^b^ |  |  |  |  |  |
| Lel | T1 | S2 | no |  | x^b^ | x^b^ |  |  |  |
| Lel | T2 | S1 | yes |  |  |  | x^b^ |  |  |
| Lel | T2 | S2^c^ | / |  |  |  |  |  |  |
| Pla | T1 | S1 | yes |  |  |  | x |  |  |
| Pla | T1 | S2 | yes |  |  |  |  |  | x |
| Pla | T1 | S3 | yes |  |  |  | x |  |  |
| Pla | T2 | S1 | no |  |  |  |  |  |  |
| Pla^a^ | T2 | S2 | no |  |  |  |  |  |  |
| Pla^c^ | T2 | S3 | no |  |  |  |  |  |  |
| PMa | T1 | S1 | no |  |  |  |  |  | x |
| PMa | T1 | S2 | yes |  |  |  | x |  | x |
| PMa | T1 | S3 | yes |  |  |  | x |  |  |
| PMaa | T2 | S1 | no |  |  |  |  |  |  |
| Pma | T2 | S2 | no |  |  |  |  | x |  |
| Pma | T2 | S3 | no |  |  |  |  |  |  |
| PS1 | T1 | S1 | yes |  |  | x |  |  | x |
| PS1 | T1 | S2 | yes |  |  | x |  |  | x |
| PS1 | T2 | S1 | yes |  |  |  | x | x |  |
| PS1 | T2 | S2 | no |  |  |  |  |  |  |
| PS2 | T1 | S1 | yes |  | x | x |  |  |  |
| PS2 | T1 | S2 | yes |  | x | x |  |  |  |
| PS2 | T2 | S1 | no |  |  |  |  | x |  |
| PS2 | T2 | S2 | no |  |  |  |  |  |  |
| Va1 | T1 | S1 | yes | x |  |  |  |  | x |
| Va1 | T1 | S2 | yes | x | x |  |  |  |  |
| Va1 | T2 | S1 | yes |  |  |  | x |  |  |
| Va1 | T2 | S2 | yes |  |  |  |  | x |  |
| Va1 | T3 | S1 | yes |  |  |  |  |  |  |

^a^site partially dried-up

^b^live stages potentially *R. dalmatina*

^c^site completely dried up^.^
